# Supplementary material for: Homotopy Phases of FQHE with Long-Range Quantum Entanglement in Monolayer and Bilayer Hall Systems
Source: Nanomaterials (Basel). 2020 Jun 30;10(7):1286. doi: 10.3390/nano10071286 (PMC7408279; doi:10.3390/nano10071286)
Supplement: Supplementary file 1 [file nanomaterials-10-01286-s001.zip › nanomaterials-814391-SM-nobib.pdf]

Supplementary Materials: Homotopy Phases of FQHE with Long-Range Quantum Entanglement in Monolayer and Bilayer Hall Systems

Janusz Edward Jacak

A. The Magnetic Field Flux Quantum in Multiply Connected Space

The magnetic field flux quantum, Φ, defines in 2D the size of a cyclotron orbit, $\frac{\Phi}{B}$. In 2D cyclotron orbits in perpendicular magnetic field are planar without the driving part along the field direction. Here we will demonstrate that the magnetic field flux quantum, Φ, has a different value in different homotopy phases. To this end one can consider the Bohr-Sommerfeld (BS) rule. This rule links the area of the 1D phase space inside the classical phase trajectory loop with the total number of quantum states corresponding to this loop. Let us write out quasi-classical wave function in a 1D well $U(x)$ with the turning points a and b,

$$\Psi(x) = \left\{ \begin{array}{ll} \frac{c}{\sqrt{p}} sin \frac{1}{\hbar} \int_a^x pdx, & \text{for } \Psi(a) = 0, \\ \frac{c'}{\sqrt{p}} sin \frac{1}{\hbar} \int_b^x pdx, & \text{for } \Psi(b) = 0, \end{array} \right. \tag{1}$$

where $p(x) = \sqrt{2m(E - U(x))}$ (to simplicity the notation, let us assume the vertical infinite borders of the well). From the uniqueness condition for the wave function one gets,

$$2\int_a^b pdx = \oint pdx = S_{xp} = n2\pi\hbar = nh, \tag{2}$$

which is the conventional formulation of BS rule (h is Planck constant, n is an integer; for the general non-vertical borders of the well $U(x)$, $S_{xp} = (n + \frac{1}{2})h$ [1]). The BS formula was derived with the condition that the trajectory is of single-loop type. However, for a distinct homotopy class with multi-loop trajectories one obtains,

$$2\int_a^b pdx = \oint pdx = S_{px} = (2k+1)n2\pi\hbar = n(2k+1)h, \tag{3}$$

for additional k loops in trajectories linking points a and b. These additional loops on both both phase trajectory branches, 'upper' $(+p)$ and 'lower' $(-p)$, together creating the closed trajectory after the integral $\oint pdx$ add $2\pi k$.

The above observation is of high significance if BS rule is applied to an effective 1D phase space (Y, P_y) of x, y components of the 2D kinematic momentum at the presence of a perpendicular magnetic field. The kinematic momentum components can be written explicitly at the Landau gauge, $\mathbf{A} = (0, Bx, 0)$ in the following form,

$$\begin{array}{l} P_x = -i\hbar\frac{\partial}{\partial x}, \\ P_y = -i\hbar\frac{\partial}{\partial y} - eBx. \end{array} \tag{4}$$

The above operators do not commute,

$$[P_x, P_y]_- = i\hbar eB. \tag{5}$$

The operators, $Y = \frac{1}{eB}P_x$ and P_y, can be thus treated as operators of canonically conjugated generalized position Y and momentum P_y, as $[Y, P_y]_- = i\hbar$. Hence, the 1D effective phase space, (Y, P_y), is actually the 2D space, (P_x, P_y). The 2D kinematic momentum space is, on the other hand, the appropriately renormalized by the factor $\frac{1}{(eB)^2}$ and turned in-plane by $\pi/2$ the ordinary 2D space (x, y). This is due

to the quasi-classical formula for the Lorentz force, $\mathbf{F} = \frac{d\mathbf{P}}{dt} = e\frac{d\mathbf{r}}{dt} \times \mathbf{B}$, which gives $dP_x = eBdy$ and $dP_y = -eBdx$ determining the relation between the trajectories in the kinematic momentum space and position space.

Now we can notice that in the 2D position space, trajectories (x, y) may in general belong to various homotopy classes with some non-contractible additional loops (as in the case of the charged particle 2D systems at sufficiently strong quantizing magnetic field). In such nontrivial trajectory homotopy case, from the BS rule one obtains,

$$S_{YP_y} = n(2k+1)h, \tag{6}$$

or rewritten to (x, y) space,

$$S_{x,y} = \frac{(2k+1)nh}{eB}, \tag{7}$$

which defines the quantum of the magnetic field flux,

$$\Phi_k = \Delta S_{x,y} B = \frac{(2k+1)h}{e}, \tag{8}$$

$\Delta S_{x,y}$ is the change of $S_{x,y}$ in Equation (7) when n is changed by 1. We see that exclusively for $k = 0$, i.e., for the homotopy class without any additional loops on trajectories, the flux quantum equals to $\Phi_0 = \frac{h}{e}$. This is the fundamental flux quantum. In other homotopy classes the flux quantum is $(2k+1)$-times greater.

Different magnetic field flux quanta Φ_k determine different sizes of the multi-loop cyclotron orbits, Φ_k/B. We have already shown that the IQHE corresponds to $k = 0$ (the homotopy class of single-loop cyclotron orbits) with the ordinary single-loop cyclotron orbit of size $\Delta S_{xy} = \frac{h}{eB_0} = \frac{S}{N} = \frac{S}{N_0}$.

The FQHE-main hierarchy line corresponds to $k = 1, 2, \ldots$ (the homotopy classes with $q = (2k+1)$-loop cyclotron orbits or braids, the halves of cyclotron orbits, with k additional loops). For example for $k = 1$ (the simplest and most pronounced Laughlin state), the three-loop cyclotron orbit is of the size $\Delta S_{xy} = \frac{3h}{eB}$. This orbit for $B = 3B_0$ perfectly fits to interparticle separation $\frac{S}{N}$ and via the commensurability condition, $\frac{3h}{eB} = \frac{S}{N}$, one gets, $\nu = \frac{N}{N_0} = \frac{N}{BSe/h} = \frac{1}{3}$.

Let us emphasize that the quasi-classical method of BS quantization used in many particle systems is interaction independent, i.e., it is accurate for arbitrarily strong interacting multiparticle systems. Thus, the sizes of the magnetic flux quanta are also interaction independent. Nevertheless, the existence of some specific homotopy trajectories in (x, y) space is conditioned by the Coulomb interaction of 2D charged particles. The interaction determines the Wigner crystal and the particle separation unavoidably required to the commensurability condition. In the hypothetical gas system of noninteracting charged particles their mutual positions can be arbitrary, which dismisses the commensurability notion and nontrivial homotopies.

The Laughlin correlations expressed by exponential, $q = 2k+1$, in the Jastrow polynomial manifesting themselves by the phase shift $q\pi$ when particle interchange [2], is the 1DUR of the cyclotron braid group generator with additional k loops [3]. Actually, for the initial 1DUR for ordinary electrons, $\sigma_i \to e^{i\pi}$, we obtain the projective 1DUR for the cyclotron subgroup at $q = 2k+1$, $\sigma_i^q \to e^{iq\pi}$, which agrees with the Laughlin phase shift $q\pi$.

B. The Degeneracy of Landau Levels

The degeneracy of LLs is the property which is independent of material and interaction and results from the form of ladder operators of kinematic momentum of a 2D charge particle (electron) exposed to a perpendicular magnetic field—it is the single-particle effect. At Landau gauge, $\mathbf{A} = [0, Bx, 0]$, $\mathbf{B} = \nabla \times \mathbf{A} = [0, 0, B]$, the kinetic energy of an electron,

$$H = -\frac{\hbar^2}{2m}\frac{\partial^2}{\partial x^2} + \frac{(-i\hbar\frac{\partial}{\partial y} - eBx)^2}{2m}, \tag{9}$$

and the wave function,

$$\Psi = e^{ip_y y/\hbar} f(x), \tag{10}$$

result in the equation for $f(x)$,

$$-\frac{\hbar^2}{2m}\frac{d^2 f(x)}{dx^2} + \frac{e^2B^2}{2m}\left(x - \frac{p_y}{eB}\right)^2 f(x) = Ef(x). \tag{11}$$

Equation (11) is the oscillator equation with energy spectrum, $E_n = (n + \frac{1}{2})\hbar\omega_B$, where $\omega_B = \frac{eB}{m}$, with the degeneracy due to a shift of the oscillator center, $x_0 = \frac{p_y}{eB}$. This oscillator center must be located inside the sample $L_x \times L_y$, i.e., $0 < x_0 < L_x$, whereas $p_y = \frac{2\pi\hbar n_y}{L_y}$. The condition for x_0 gives,

$$0 < p_y < eBL_x, \tag{12}$$

thus the total number of states for a whole range of p_y equals to $\frac{L_x L_y Be}{h}$, which is the degeneracy of LLs, N_0.

A filling rate of LLs is expressed by the ratio $\nu = \frac{N}{N_0}$. In the case of Chern topological insulators without LLs, a filling rate is counted per crystalline node, i.e., $\nu = \frac{N}{n_0}$, where n_0 is number of nodes in the lattice.

C. Reentrant IQHE in a Single Hall Layer

The problem of so-called reentrant IQHE experimentally observed in a single layer of a conventional 2DES or graphene manifests itself as quantization $R_{xy} = \frac{h}{e^2\nu}$ with $\nu = 1, 3, \ldots$, and $R_{xx} = 0$ but for an actual filling ν_{nom} deviated from integer ν. This deviation is quite large, and the reentrant behavior is observed in GaAs samples even up to $\nu_{nom} \in (0.8, 1.2)$. It is easy to explain this effect as the result of the energy competition enforcing a local redistribution of electrons in such a way that electrons are concentrated in stripes with local filling rate exactly equal to $\nu_{local} = 1$ (or other integers for higher LLs). The integer local filling in stripes allows for formation of IQHE correlations ranged to stripes despite the fractional nominal filling. This is possible because the energy gain due to IQHE state formation in stripes prevails the increase of the Coulomb interaction energy due to striping when electrons attain local density beyond or below the actual charge density of the jellium. The simple simulation of the related energy trade-off is as follows (ΔE per the area l_B^2, in units of $\frac{1}{4\pi\epsilon_0}\frac{e^2}{l_B}$),

$$\begin{aligned} \Delta E = &-\tfrac{l-x}{l(2\pi)^2}\int_0^l dx_1 \int_x^l dx_2 \int_0^L dy_1 \int_0^L dy_2 \frac{1}{\epsilon_1\sqrt{(x_1-x_2)^2+(y_1-y_2)^2}} \\ &+ \left(\tfrac{l-x}{l2\pi}\right)^2 \int_0^l dx_1 \int_0^l dx_2 \int_0^L dy_1 \int_0^L dy_2 \frac{1}{\epsilon_1\sqrt{(x_1-x_2)^2+(y_1-y_2)^2}} \\ &+0.85 \times 0.5 \left(\tfrac{l}{2\pi}\right)^2 \int_x^l dx_1 \int_x^l dx_2 \int_0^L dy_1 \int_0^L dy_2 \frac{1}{\epsilon_1\sqrt{(x_1-x_2)^2+(y_1-y_2)^2}} \\ &-0.92 \times 0.5 \left(\tfrac{l-x}{2l\pi}\right)^2 \int_0^l dx_1 \int_0^l dx_2 \int_0^L dy_1 \int_0^L dy_2 \frac{1}{\epsilon_1\sqrt{(x_1-x_2)^2+(y_1-y_2)^2}}, \end{aligned} \tag{13}$$

where integrations account for the Coulomb energy of attraction between electrons either located in the stripe $(l-x) \times L$ with $\nu_{local} = 1$ or on the entire plaquette with $\nu_{nom} = \frac{l-x}{l}$ and the jellium of size $l \times L$ positively charged with the density per l_B^2, $\rho = \frac{l-x}{l2\pi}$ (size dimensions are expressed in units of l_B, the energy in units of $\frac{1}{4\pi\epsilon_0}\frac{e^2}{l_B}$). The interaction between electrons in the stripe with $\nu_{local} = 1$ is assessed according to Metropolis Monte Carlo simulation [4] of IQHE (cf. Supplementary Materials E). This procedure results in the coefficient 0.85 reducing electron interaction in Laughlin-type state with the Jastrow polynomial with $q = 1$ (IQH state). The electrons compressed in the stripe $(l-x) \times L$ are not balanced by the local jellium with lower charge density $\frac{l-x}{l}\frac{1}{2\pi}$ corresponding to the nominal filling rate $\nu_{nom} = \frac{l-x}{l}$. The energy gain due to incompressible IQHE state in the stripe prevails; however, the electrostatic energy increase is caused by imbalanced charge distribution. The plot for ΔE assessed by the Monte Carlo simulation is presented in Figure S1 (for $l = L = 100(500)l_B$ and $\varepsilon_1 = 12.9(2)$ for

GaAs and graphene, respectively). One can notice that reentrant IQHE is energetically convenient for $x < x^*$ ($\Delta E(x^*) = 0$), which gives (for considered in the model $l = L = 100(500)l_B$) $\nu_{nom} > 0.82$ (for graphene with four times lower permittivity in free space [11] the reentrant deviation in energy is larger, but the onset of reentrant IQHE is similar for GaAs $\nu_{nom} > 0.8$. We thus conclude that the constant 0.92 in Equation (13) properly models the uncorrelated state contribution for the IQHE reentrance, and this factor is next used in Equations (6) and (7).

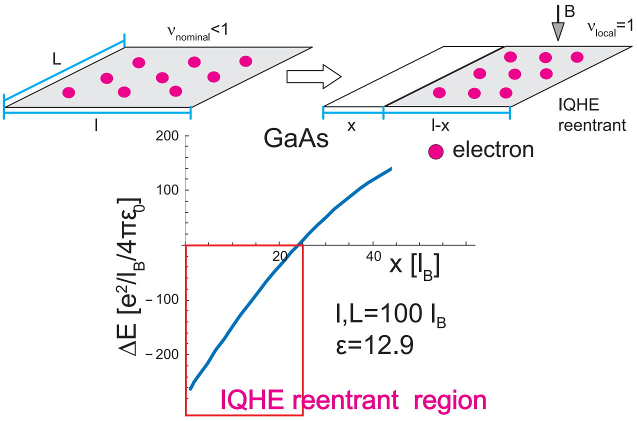

Figure S1. (**Upper**) The pictorial illustration of the shift of electron distribution resulting in reentrant IQHE. (**Lower**) Evaluation of ΔE according to Equation (13) versus x, $\nu_{nominal} = \frac{l-x}{l}$ (for $x = 20l_B$ and $l = 100l_B$, $\nu_{nominal} = 0.8$ as is visible in experiment [12]).

To assess the energy of electron-electron interaction it has been used the Metropolis Monte Carlo simulation method [4] (cf. Supplementary Materials E), which allows for calculation of the e-e interaction energy averaged over the correlated quantum multiparticle state given by the Laughlin function or (as needed in our case) by the Slater function for completely filled the lowest LL (for $\nu = 1$), $\left\langle \Psi(z_1,\ldots,z_N) \left| \sum_{i>j}^{N} \frac{1}{|z_i - z_j|} \right| \Psi(z_1,\ldots,z_N) \right\rangle$ (here $z_l = x_l + iy_l$ are complex representations of particle positions $\mathbf{r}_l = (x_l, y_l)$, $l = 1,\ldots,N$). This energy per single-particle scales with N as proportional to $\sqrt{N}$, cf. Figure S3. Similarly scales the energy of Coulomb interaction jellium-jellium, which can be accounted for analytically (in cylindrical geometry), $\frac{\rho^2}{2} \int_S d^2r_1 \int_S d^2r_2 \frac{1}{|\mathbf{r}_1 - \mathbf{r}_2|} = \frac{8\sqrt{\nu}\sqrt{N}}{3\pi\sqrt{2}}$. The energy of interaction of electrons with jellium averaged over the correlated Hall state,

$$\left\langle \Psi(z_1,\ldots,z_N) \left| -\rho \sum_{i=1}^{N} \int_S d^2r \frac{1}{|\mathbf{r} - \mathbf{r}_i|} \right| \Psi(z_1,\ldots,z_N) \right\rangle /N,$$

can be also assessed by the Metropolis Monte Carlo approach for quantum averaging, using the analytical form of the integral, $\rho \int_S d^2r \frac{1}{|\mathbf{r}-\mathbf{r}_i|} = \sqrt{2\nu}\sqrt{N} f\left(\frac{|\mathbf{r}_i|}{R}\right)$, with $f(x) = \begin{cases} \frac{2E(x)}{\pi}, & x \leq 1 \\ \left(\frac{1}{2x}\right) F_{2;1}(1/2, 1/2 : 2 : 1/x^2), & x \geq 1 \end{cases}$, where $E(x)$ is the complete elliptic integral and $F_{2;1}(a, b : c : y)$ is the hypergeometric function (R is the radius of the sample plaquette in cylindrical geometry). The scaling of the averaged energy of electron-jellium interaction as proportional to $\sqrt{N}$ is explicit.

D. Energy Trade-Off in Bilayer Hall System—Contributions to Equation (11) [Main Text]

The terms $\Delta E_{top(bot)}$ in Equation (11) [Main Text] represent the energy gain caused by the striping internally in each layer, top and bottom, respectively. The term ΔE_{inter} represents the contribution to the energy gain due to the inter-layer interaction. They have the following form,

$$\begin{aligned} \Delta E_{top} &= el_{top}\left[\nu_{local}=1\right] \leftrightarrow jell_{top}\left[\rho=\tfrac{l-x}{l2\pi}\right] \\ &- el_{top}\left[\nu_{nom}=\tfrac{l-x}{l}\right] \leftrightarrow jell_{top}\left[\rho=\tfrac{l-x}{l2\pi}\right] \\ &+ el_{top}\left[\nu_{local}=1\right] \leftrightarrow el_{top}\left[\nu_{local}=1\right] \\ &- el_{top}\left[\nu_{nom}=\tfrac{l-x}{l}\right] \leftrightarrow el_{top}\left[\nu_{nom}=\tfrac{l-x}{l}\right] \end{aligned} \tag{14}$$

and

$$\begin{aligned} \Delta E_{bot} &= el_{bot}\left[\nu_{local}=1\right] \leftrightarrow jell_{bot}\left[\rho=\tfrac{x}{l2\pi}\right] \\ &- el_{bot}\left[\nu_{nom}=\tfrac{x}{l}\right] \leftrightarrow jell_{bot}\left[\rho=\tfrac{x}{l2\pi}\right] \\ &+ el_{bot}\left[\nu_{local}=1\right] \leftrightarrow el_{bot}\left[\nu_{local}=1\right] \\ &- el_{bot}\left[\nu_{nom}=\tfrac{x}{l}\right] \leftrightarrow el_{bot}\left[\nu_{nom}=\tfrac{x}{l}\right]. \end{aligned} \tag{15}$$

In the above expressions, we used the following notation: $\leftrightarrow$ marks the Coulomb interaction between electrons (*el*) or jellium (*jell*) in a single layer (*top* or *bottom*) in the stripes with the indicated local filling factors adjusted to the presence or absence of the striped structure. The last term in Equation (11) [Main Text] describes the energy gain due to the inter-layer interaction across the barrier in the stripe structure. It has the form,

$$\begin{aligned} \Delta E_{inter} &= el_{top}\left[\nu_{local}=1\right] \leftrightarrow jell_{bot}\left[\rho=\tfrac{x}{l2\pi}\right] \\ &- el_{top}\left[\nu_{nom}=\tfrac{l-x}{l}\right] \leftrightarrow jell_{bot}\left[\rho=\tfrac{x}{l2\pi}\right] \\ &+ el_{bot}\left[\nu_{local}=1\right] \leftrightarrow jell_{top}\left[\rho=\tfrac{l-x}{l2\pi}\right] \\ &- el_{bot}\left[\nu_{nom}=\tfrac{x}{l}\right] \leftrightarrow jell_{top}\left[\rho=\tfrac{l-x}{l2\pi}\right] \\ &+ el_{top}\left[\nu_{local}=1\right] \leftrightarrow el_{bot}\left[\nu_{local}=1\right] \\ &- el_{top}\left[\nu_{nom}=\tfrac{l-x}{l}\right] \leftrightarrow el_{bot}\left[\nu_{nom}=\tfrac{x}{l}\right]. \end{aligned} \tag{16}$$

The expressions (14), (15) and (16) have the following explicit forms, respectively,

$$\begin{aligned} \Delta E_{top} = &- \tfrac{l-x}{l(2\pi)^2}\int_x^l dx_1\int_0^L dy_1\int_0^l dx_2\int_0^L dy_2\mathcal{A}(1,2) \\ &+ \left(\tfrac{l-x}{l2\pi}\right)^2\int_0^l dx_1\int_0^L dy_1\int_0^l dx_2\int_0^L dy_2\mathcal{A}(1,2) \\ &+ 0.85\times 0.5\left(\tfrac{1}{2\pi}\right)^2\int_x^l dx_1\int_0^L dy_1\int_x^l dx_2\int_0^L dy_2\mathcal{A}(1,2) \\ &- 0.92\times 0.5\left(\tfrac{l-x}{l2\pi}\right)^2\int_0^l dx_1\int_0^L dy_1\int_0^l dx_2\int_0^L dy_2\mathcal{A}(1,2), \end{aligned} \tag{17}$$

$$\begin{aligned} \Delta E_{bot} = &- \tfrac{x}{l(2\pi)^2}\int_0^x dx_1\int_0^L dy_1\int_0^l dx_2\int_0^L dy_2\mathcal{A}(1,2) \\ &+ \left(\tfrac{x}{l2\pi}\right)^2\int_0^l dx_1\int_0^L dy_1\int_0^l dx_2\int_0^L dy_2\mathcal{A}(1,2) \\ &+ 0.85\times 0.5\left(\tfrac{1}{2\pi}\right)^2\int_0^x dx_1\int_0^L dy_1\int_0^x dx_2\int_0^L dy_2\mathcal{A}(1,2) \\ &- 0.92\times 0.5\left(\tfrac{x}{l2\pi}\right)^2\int_0^l dx_1\int_0^L dy_1\int_0^l dx_2\int_0^L dy_2\mathcal{A}(1,2), \end{aligned} \tag{18}$$

$$
\begin{aligned}
\Delta E_{inter} = & - \frac{x}{l(2\pi)^2}\int_x^l dx_1 \int_0^L dy_1 \int_0^l dx_2 \int_0^L dy_2 \mathcal{B}(1,2) \\
& + \frac{x(l-x)}{(l2\pi)^2}\int_0^l dx_1 \int_0^L dy_1 \int_0^l dx_2 \int_0^L dy_2 \mathcal{B}(1,2) \\
& - \frac{l-x}{l(2\pi)^2}\int_0^x dx_1 \int_0^L dy_1 \int_0^l dx_2 \int_0^L dy_2 \mathcal{B}(1,2) \\
& + \frac{x(l-x)}{(l2\pi)^2}\int_0^l dx_1 \int_0^L dy_1 \int_0^l dx_2 \int_0^L dy_2 \mathcal{B}(1,2) \\
& + \frac{1}{(2\pi)^2}\int_x^l dx_1 \int_0^L dy_1 \int_0^x dx_2 \int_0^L dy_2 \mathcal{B}(1,2) \\
& - \frac{x(l-x)}{(l2\pi)^2}\int_0^l dx_1 \int_0^l dy_1 \int_0^l dx_2 \int_0^l dy_2 \mathcal{B}(1,2),
\end{aligned} \tag{19}
$$

in the above, $\mathcal{A}(1,2) = \frac{1}{\varepsilon_1\sqrt{(x_1-x_2)^2+(y_1-y_2)^2}}$, $\mathcal{B}(1,2) = \frac{1}{\varepsilon_2\sqrt{d^2+(x_1-x_2)^2+(y_1-y_2)^2}}$, $\varepsilon_{1(2)}$ denotes the dielectric permittivity of the layer (barrier) material. All dimensions are taken in l_B units.

In Equation (11) [Main Text], ΔE means the energy gain of the striped structure with local $\nu_{local} = 1$ in the filled stripes with respect to the non-striped uniform two layers with the nominal $\nu_{bot} = \frac{x}{l}$ and $\nu_{top} = \frac{l-x}{l}$ ($\nu_{bot} + \nu_{top} = 1$) for an arbitrary $x \in (0, l)$. One can notice that the condition $\nu_T = 1$ (or other integer) is connected with the geometrically unique situation when two stripes in the top layer (the first one of width x and empty of electrons (positively charged due to jellium) while the subsequent one of $l - x$ width overfilled with electrons up to local $\nu_{local} = 1$) ideally fit to the inverted stripe ordering in the bottom layer, as shown in Figure S2. The negatively charged stripes are over-charged with electrons (beyond the positive jellium charge still corresponding to the nominal filling rate $\nu_{top} = \frac{l-x}{l}$ and $\nu_{bot} = \frac{x}{l}$) up to local $\nu_{local} = 1$ (with the density of electrons $\frac{1}{2\pi}$ per l_B^2 surface, and the jellium charge density $\frac{\nu_{nominal}}{2\pi}$).

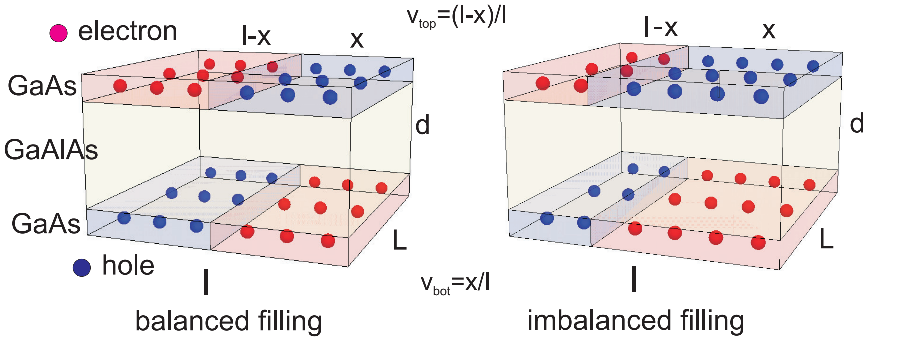

Figure S2. The cartoon presentation of the bilayer Hall structure GaAs/GaAlAs/GaAs with marked dimension of an elementary striped sector of length l. Electrons are shifted to the over-charged stripes, while empty stripes are hole stripes charged by the positive jellium. The structure is mirrored symmetrical with charge change across the barrier of thickness d, which is possible only for a complementary filling of layers, $\nu_{top} + \nu_{bot} = 1$ (r other integer), with $\nu_{top} = \frac{(l-x)}{l}$ and $\nu_{bot} = \frac{x}{l}$ (in left panel, both equal to 1/2).

Two last multi-fold integrals in Equations (17) and (18) describe the quantum effects beyond the electrostatic interaction of stripes and are crucial for the total energy trade-off and the resulting phase diagrams. The factors 0.85, 0.92 in these integrals correspond to the energy gain due to electron-electron interaction in the correlated IQH state (0.85) for the $\nu_{local} = 1$ with respect to not correlated state at $\nu_{nominal} < 1$ (the latter entering with the factor 0.92). The factors, 0.85 and 0.92, are most important for the energy minimization and are determined by the Monte Carlo Metropolis method [4] used to the estimation of the energy of correlated integer quantum Hall state with respect to the uncorrelated state and adjusted (0.92) to the experimental data for the observed range of a monolayer reentrant IQHE state ($\nu_{reentrant}^{IQH} \in (0.8, 1.2)$). The factors for the IQH state in the overfilled stripes (0.85) is assessed by the numeric Monte Carlo Metropolis method [4] for the multiparticle Slater-type wave function for IQHE [2].

E. Metropolis Monte Carlo Assessment of the Energy Gain Due to IQHE Correlations

The method has been developed [4] to assess multidimensional of high order integrals for particle interaction energy in states described by the Laughlin function. Instead of taking the multidimensional integral directly, a quickly convergent iteration random procedure is applied to define the particle distribution governed by the density of probability given by the multiparticle wave function under analysis. The repeating small finite and random steps for position changes of all particles are undertaken up to determination of a maximal value of the wave function modulus at final positions. The iteration of this procedure repeated ca. 10^8 times defines the distribution of particle positions in agreement with the multiparticle wave function, in this case, with the Laughlin functions for FQHE states (including the IQHE state for $\nu = 1$, when the Jastrow polynomial has the Vandermonde determinant form). The resulted distribution of particle positions is independent of a start points of the iteration and reveals interparticle correlations specific to a particular quantum Hall state. The determination of different but equivalent distributions of particle positions is repeated next ca. 10^6 times densely covering local maxima of the examined multiparticle wave function. Each distribution allows for estimation of the corresponding electron-electron and electron-jellium interactions in accordance with the multiparticle wave function. Next, these particular results for the interaction energies are averaged over all 10^6 repetitions of particle distribution determination which finally gives the quantumly averaged the electron-electron and electron-jellium interaction energies with high accuracy confirmed by the perfect consistence with the exact diagonalization on small models and with the experimentally measured activation energies for states at various filling rates. The method is efficient and relatively quick and not limited to small N only (contrary to the exact diagonalization of the interaction feasible only in very small model Hall systems). Therefore, this method is especially convenient and sufficiently accurate to study an effect of the reentrant IQHE in twin Hall structures, where inclusion of relatively high number of electrons is necessary.

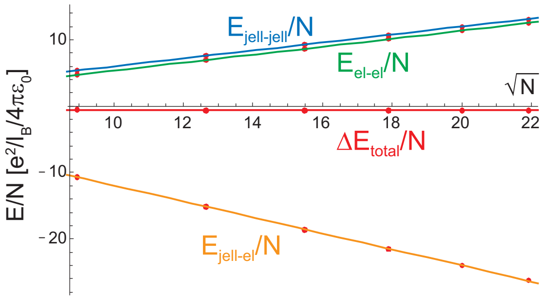

Figure S3. The components of the interaction energy per single particle for the correlated state of IQHE, $\nu = 1$ as listed in Table S1, versus $\sqrt{N}$ (by Metropolis Monte Carlo method of averaging over quantum states given by the multiparticle Laughlin-type wave function with $q = 1$ for $\nu = 1$).

Table S1. Energies of various Coulomb channels for $\nu = 1$ IQH state calculated by Metropolis Monte Carlo simulation (in units of $\frac{e^2}{l_B}\frac{1}{4\pi\varepsilon_0}$).

N	$E_{jell-jell}/N$	$E_{jell-el}/N$	E_{el-el}/N	$\Delta E_{total}/N$
80	5.36845	−10.6326	4.6936	−0.570516
160	7.59213	−15.1761	6.95414	−0.629868
240	9.29843	−18.5908	8.672	−0.620415
320	10.7369	−21.4734	10.1046	−0.631899
400	12.0042	−24.0026	11.3821	−0.616286
480	13.15	−26.2682	12.4993	−0.618932

In more details, the Metropolis Monte Carlo method [4] consists of an assessment of the quantum mean value,

$$\langle\Psi_m(z_1,\ldots,z_N)\,|A(z_1,\ldots,z_N)|\,\Psi_m(z_1,\ldots,z_N)\rangle, \tag{20}$$

for $\Psi(z_1,\ldots,z_N)=\prod_{i<j}^{N}(z_i-z_j)^q e^{\left[-\sum_{i=1}^{N}|z_i|^2/4l_B^2\right]}$. The function $A(z_1,\ldots,z_N)$ may be averaged over numerous (of 10^6 order) various distributions of $z_1,\ldots,z_N$ satisfying the detailed balance according to the probability density $\langle\Psi|\,\Psi\rangle$. Such particular particle position distributions are determined by the random walk for all variables $z_1,\ldots,z_N$ starting from the randomly selected initial start points with the positive feedback imposed assuring a growth of the probability density $\langle\Psi|\,\Psi\rangle$ along the random walk. This gives the convergence to an optimal distribution of particle positions respecting correlations involved in the Laughlin wave function. The repetition of this procedure sufficiently many times and the averaging of the quantity $A(z_1,\ldots,z_N)$ over all distributions efficiently and quickly approaches searched quantum mean value of A. The procedure is the subject of its optimization by the control of the feedback criteria and the length of steps of the random walk of point positions [4].

The Metropolis MC procedure may be applied to Hall systems with varying particle number N. Next, the results for averaged energies can be extrapolated to the thermodynamic limit (due to scaling of all multiparticle averaged energies with respect to $\sqrt{N}$). In Table S1 there are collected a few exemplary energies related to the Hall system with respect to N for the IQHE state, $\nu=1$ (cf. Figure S3). From these data one can refine the coefficient 0.85 used in Equations (6) and (7) as well as in Equation (13) for representation of the $el-el$ interaction energy in IQHE phase in the integral form, $0.85\times\frac{1}{2}\left(\frac{l}{2\pi}\right)^2\int_x^l dx_1\int_x^l dx_2\int_0^L dy_1\int_0^L dy_2\frac{1}{\epsilon_1\sqrt{(x_1-x_2)^2+(y_1-y_2)^2}}$, in the plaquette $(l-x)\times L$ with local filling $\nu_{local}=1$.

F. Two-Component Bogolubov Model for Superfluid Exciton BEC in Bilayer Hall Systems

Creation of the indirect excitons in a twin Hall system is related with the inter-layer Coulomb coupling of the LL band electrons and holes occupying appropriate complementary states in opposite layers numbered by the LL quantum numbers $(k,n=0)$ in the LLL, as given by Equation (10) [Main Text] (in the Landau gauge $A=(0,Bx,0)$).

The striping in k-space corresponds to the striping of electron distribution in $x\times y$ space complementary in opposite layers and can be accounted for by the similar electrostatic energy gain as given in Equation (11) [Main Text]. Striping in k-space may in particular correspond to a longitudinal or transverse orientation of stripes depending on the selected gauge. The arbitrariness of the gauge choice allows for maintenance of the k striping in the counterflow configuration.

The two-component, $(\pm)$ and $(\mp)$, BEC of the indirect excitons with suppressed k number (enumerating the LL degeneracy of states given by Equation (10)) [Main Text] may be described in terms of a new quantum number—2D momentum p [5,6] via the model Hamiltonian,

$$\begin{aligned} H &= H^a+H^b\\ &=\sum_p E_p^a a_p^+a_p+\frac{1}{2S}\sum_{p_1,p_2,p_3,p_4,p_1+p_2=p_3+p_4} u_a(|p_1-p_4|)a_{p_1}^+a_{p_2}^+a_{p_3}a_{p_4}\\ &+\sum_p E_p^b b_p^+b_p+\frac{1}{2S}\sum_{p_1,p_2,p_3,p_4,p_1+p_2=p_3+p_4} u_b(|p_1-p_4|)b_{p_1}^+b_{p_2}^+b_{p_3}b_{p_4}, \end{aligned} \tag{21}$$

where $a(b)_p^{(+)}$ are the boson–exciton annihilation (creation) operators in p state for $\pm(\mp)$ polarization; $E_p^{a(b)}=\frac{p^2}{2m^*_{a(b)}}$ is the kinetical energy with the effective mass $m^*_{a(b)}$ of the exciton (as derived in [5], the mass of exciton in Hall system is not connected to band characteristics, and the current direction with respect to the external electric field is governed by the drift scheme [5,7–10]). In this way we model the two-component indirect exciton liquid consisting of oppositely polarized electron-hole pairs suppressing their internal structure, though the momentum p of excitons may be associated, at selected Landau gauge, with the pairing of $(k+p/2,1(2),-k+p/2,2(1))$ electron-hole states [5,7–

10]. In the effective Hamiltonian (21) we neglect the interaction between differently polarized excitons because their spatial separation in k stripes, though interaction between stripes has been included by appropriate electrostatic terms in Equation (11) [Main Text]. A difference in population of oppositely polarized excitons displays an imbalance of filling rates, $\nu_T = \nu_{bot} + \nu_{top}$. The initial BEC is associated in the model (21) with the condensate of a macroscopic number $N_0^{a(b)}$ of bosons $a(b)_p^+$ in $p = 0$ state ($N_0^{a(b)} \simeq N^{a(b)}$, at low temperatures, $N^{a(b)}$—total number of excitons with polarization $\pm(\mp)$ in the system). We henceforth suppress the further analysis to the only one subsystem as the second one is analogous. Because $a_0^+ a_0 |0\rangle = N^a |0\rangle$, where $|0\rangle$ is the ground state ($T = 0$), for large N_0^a one can approximate operators by numbers, $a_0^+ = a_0 = \sqrt{N_0^a}$, and then,

$$\begin{aligned} H^a &\simeq \frac{1}{2S} u(0) N_0^{a2} + \sum_p \left[E_p^a + \frac{N_0^a}{S} u_a(|p|) \right] a_p^+ a_p \\ &+ \frac{N_0^a}{2S} \sum_{p \neq 0} u_a(|p|) (a_p^+ a_{-p}^+ + a_p a_{-p}). \end{aligned} \tag{22}$$

After the Bogolubov diagonalization, $a_p = \alpha(p)\tilde{a}_p + \beta^*(p)\tilde{a}_{-p}^+$, $a_p^+ = \alpha^*(p)\tilde{a}_p^+ + \beta(p)\tilde{a}_{-p}$, one finds,

$$H^a \simeq \frac{1}{2S} u_a(0) N_0^{a2} + \Delta E^a + \sum_p \epsilon_p^a \tilde{a}_p^+ \tilde{a}_p, \tag{23}$$

where $\epsilon_p^a = \sqrt{\frac{p^4}{4m_a^{*2}} + \frac{N_0^a p^2 u_a(|p|)}{m_a^* S}} = |p| \sqrt{\frac{p^2}{4m_a^{*2}} + \frac{N_0^a u_a(|p|)}{m_a^* S}}$ (and analogously for H^b part of the Hamiltonian (21)). For the long-wave-length limit (small $|p|$), $\epsilon_p^a \simeq c^a |p|$, with the constant $c^a = \sqrt{\frac{N_0^a u_a(0)}{m_a^* S}}$ is the phonon-type spectrum provided that $u_a(0) > 0$, i.e., the original bosons must repulse themselves; $\Delta E^a = -\frac{1}{2} \sum_{p,p \neq 0} (E_k^a + u_a(|p|) \frac{N_0^a}{S} - \epsilon_p^a)$. Hence, for the repulsing bosons the low energy excitation spectrum beyond the BEC is of phonon-type, which guarantees the superfluidity of the BEC due to impossibility to simultaneously fulfill a conservation of the energy and momentum for such phonon-type excitations at scatterings [6]. We see that $c^a \sim \sqrt{N_0^a}$ but in the twin Hall configuration with $\nu_T = \nu_{top} + \nu_{bot} = 1$ we deal with $N_0^a = \nu_{bot} \frac{S}{2\pi}$, whereas $N_0^b = \nu_{top} \frac{S}{2\pi}$ and $N_0^a + N_0^b = \frac{S}{2\pi}$ (S is the single layer surface in l_B^2 units). In the balanced case $u_a(0) \simeq u_b(0)$, but the imbalanced filling, $N_0^a \neq N_0^b$, causes difference in superfluid properties of both components of exciton liquid. The critical superflow, $j_{a(b)} = \frac{\nu_{bot(top)}}{2\pi} c^{a(b)}$, is lower for smaller filling $\nu_{top(bot)}$ also due to lower $c^{a(b)} \sim \sqrt{N_0^{a(b)}}$. The drift velocity, perpendicular to the applied electric field E, is $\sim \frac{E}{B}$, thus the critical electric field $E_c \simeq B c^{a(b)}$ and differs for opposite polarizations at the imbalanced filling of the twin system.

In the model (21), the operators $a^+(b^+)_p$ are ideal boson operators, whereas the initial indirect excitons—let us represent them by operators labeled with k, $a^+(b^+)_k$, are not exact bosons due to their internal structure expressed in k states (Equation (10) [Main Text]), as $a_k^+ a_k^+ = 0$ ($b_k^+ b_k^+ = 0$). The applicability of the BEC model is here conditioned by the degeneracy of exciton k states, which results from the LL massive degeneracy. Due to this degeneracy the quasi-boson excitons, $a^+(b^+)_k$, with the same energy may be modeled by the BEC in the $p = 0$ state of true bosons, $a^+(b^+)_p$, experiencing the magnetic field presence only by their internal structure [5,7,8,10] and weakly repulsing due to k striping, which leads to their superfluidity. Please note that k enumerates states given by Equation (10) [Main Text] in the main text and marks the internal structure of excitons at an assumed gauge, but not the energy (neither of electrons and of holes nor of excitons) and, moreover, may be substituted by a distinct quantum number if one changes the gauge of the magnetic field potential. The momentum p of the exciton is thus irrelevant to k (and to gauge) [5,7–10] though the role of the k striping is essential for the exciton BEC superfluidity.

The striping in k-space corresponds to the complementary striping of electron distribution in $x \times y$ space in both layers and can be accounted in the simulation as was presented in the main text. Striping in k-space may correspond to the distinct stripe orientation in $x \times y$ space depending on

the selected gauge. The arbitrariness of the gauge choice allows for k striping maintenance in the counterflow configuration in agreement with the experimental observations.

The defined above model of the two-component BEC of indirect excitons with two opposite polarizations, $(\pm)$ and $(\mp)$, agrees with the theory developed in Refs [5,7–10], where the low energy spectrum for Landau quantized electron systems in twin layers separated by an insulating barrier has been discussed assuming the ground state in the form of the BEC of indirect excitons. For limiting situation of a zero thickness of the barrier for excitons (neglecting their instability due to exciton recombination) the parabolic low energy dispersion above the BEC has been derived precluding the BEC superfluidity in a Hall monolayer. However, for a phonon-type excitation dispersion, found for a finite thickness of the dielectric barrier [9,10], the superfluidity is admitted. The latter, though limited to a single $\mp$ polarization of excitons in Refs [9,10], actually proves the repulsion of the indirect excitons in the considered two-component model (21).

References

1. Landau, L.; Liifsitz, E.M. *Quantum mechanics, no-relativistic theory*; Pergamon Press: Oxford, 1965.
2. Laughlin, R.B. Anomalous quantum Hall effect: an incompressible quantum fluid with fractionally charged excitations. *Phys. Rev. Lett.* **1983**, *50*, 1395.
3. Jacak, J.; Gonczarek, R.; Jacak, L.; Jóźwiak, I. *Application of Braid Groups in 2D Hall System Physics: Composite Fermion Structure*; World Scientific: Singapore, 2012.
4. Ciftja, O.; Wexler, C. Monte Carlo simulation method for Laughlin-like states in a disk geometry. *Phys. Rev. B* **2003**, *67*, 075304.
5. Gorkov, L.P.; Dzyaloshinskii, I.E. Contribution to the theory of the Mott exciton in a strong magnetic field. *Sov. Phys. JETP* **1968**, *26*, 449.
6. Bogolubov, N.N. On the theory of supefluidity. *Journal of Phys.* **1947**, *11*, 23.
7. Paquet, D.; Rice, T.M.; Ueda, K. Two-dimensional electron-hole fluid in a strong perpendicular magnetic field: Exciton Bose condensate or maximum density two-dimensional droplet. *Phys. Rev. B* **1985**, *32*, 5208.
8. Kallin, C.; Halperin, B. Excitations from a filled Landau level in the two-dimensional electron gas. *Phys. Rev. B* **1984**, *30*, 5655.
9. MacDonald, A. Superfuid properties of double-layer quantum Hall ferromagnets. *Phys. E* **2001**, *298*, 129.
10. Fertig, H.A. Energy spectrum of a layered system in a strong magnetic field. *Phys. Rev. B* **1989**, *40*, 1087.
11. Santos, E.; Kaxiras, E. Electric-Field Dependence of the Effective Dielectric Constant in Graphene. *Nano Lett.* **2013**, *13*, 898.
12. Pan, W.; Störmer, H.L.; Tsui, D.C.; Pfeiffer, L.N.; Baldwin, K.W.; West, K.W. Fractional quantum Hall effect of composite fermions. *Phys. Rev. Lett.* **2003**, *90*, 016801.
