# Supplementary figures and images for: Homotopy Phases of FQHE with Long-Range Quantum Entanglement in Monolayer and Bilayer Hall Systems
Source: Nanomaterials (Basel). 2020 Jun 30;10(7):1286. doi: 10.3390/nano10071286 (PMC7408279; doi:10.3390/nano10071286)

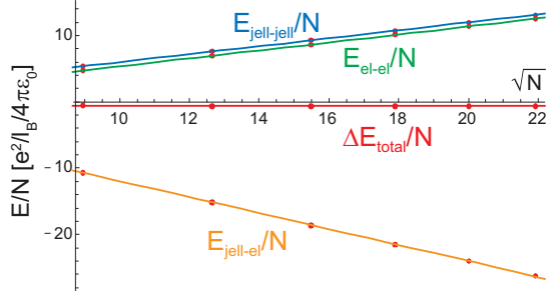

Supplement: Supplementary file 1 [file nanomaterials-10-01286-s001.zip › Definitions/fig-SI-13-eps-converted-to.pdf]

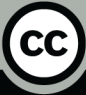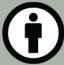

BY

Supplement: Supplementary file 1 [file nanomaterials-10-01286-s001.zip › Definitions/logo-ccby-eps-converted-to.pdf]

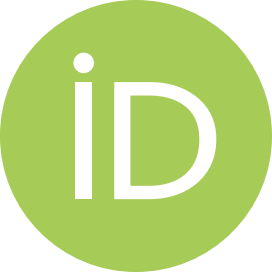

Supplement: Supplementary file 1 [file nanomaterials-10-01286-s001.zip › Definitions/logo-orcid-eps-converted-to.pdf]

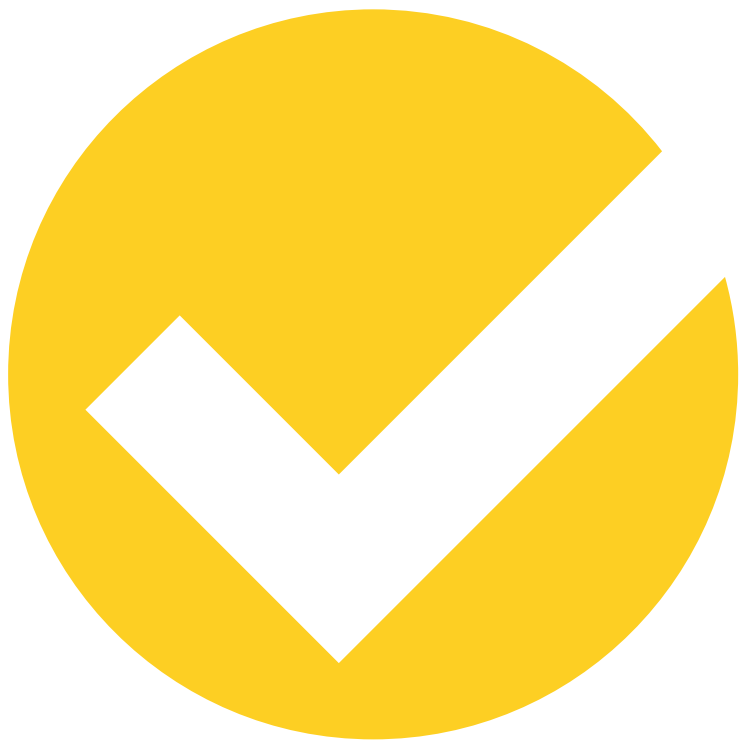

check for  
updates

Supplement: Supplementary file 1 [file nanomaterials-10-01286-s001.zip › Definitions/logo-updates.pdf]

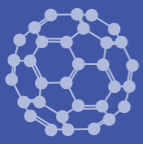

*nanomaterials*

Supplement: Supplementary file 1 [file nanomaterials-10-01286-s001.zip › Definitions/nanomaterials-logo-eps-converted-to.pdf]

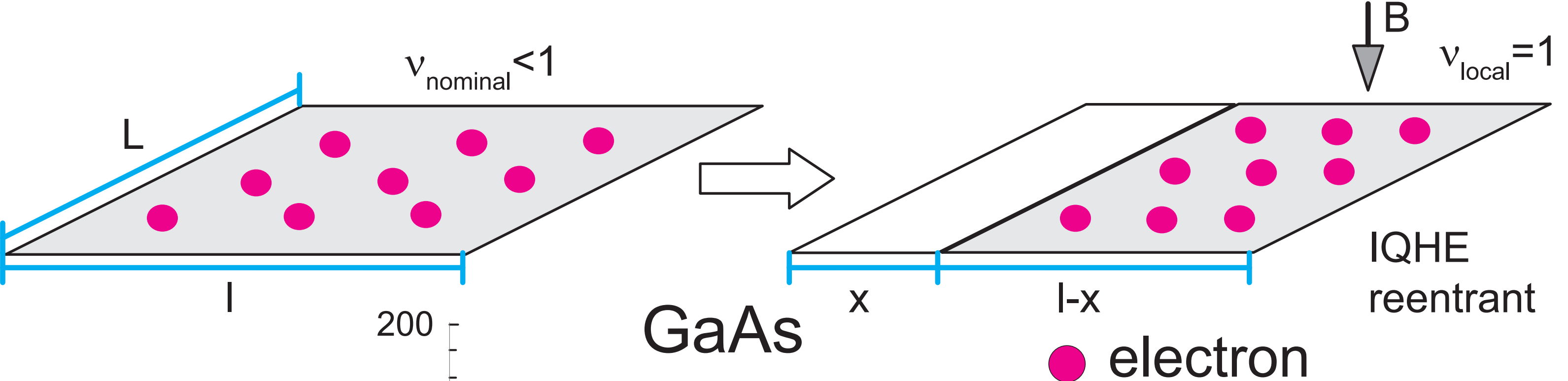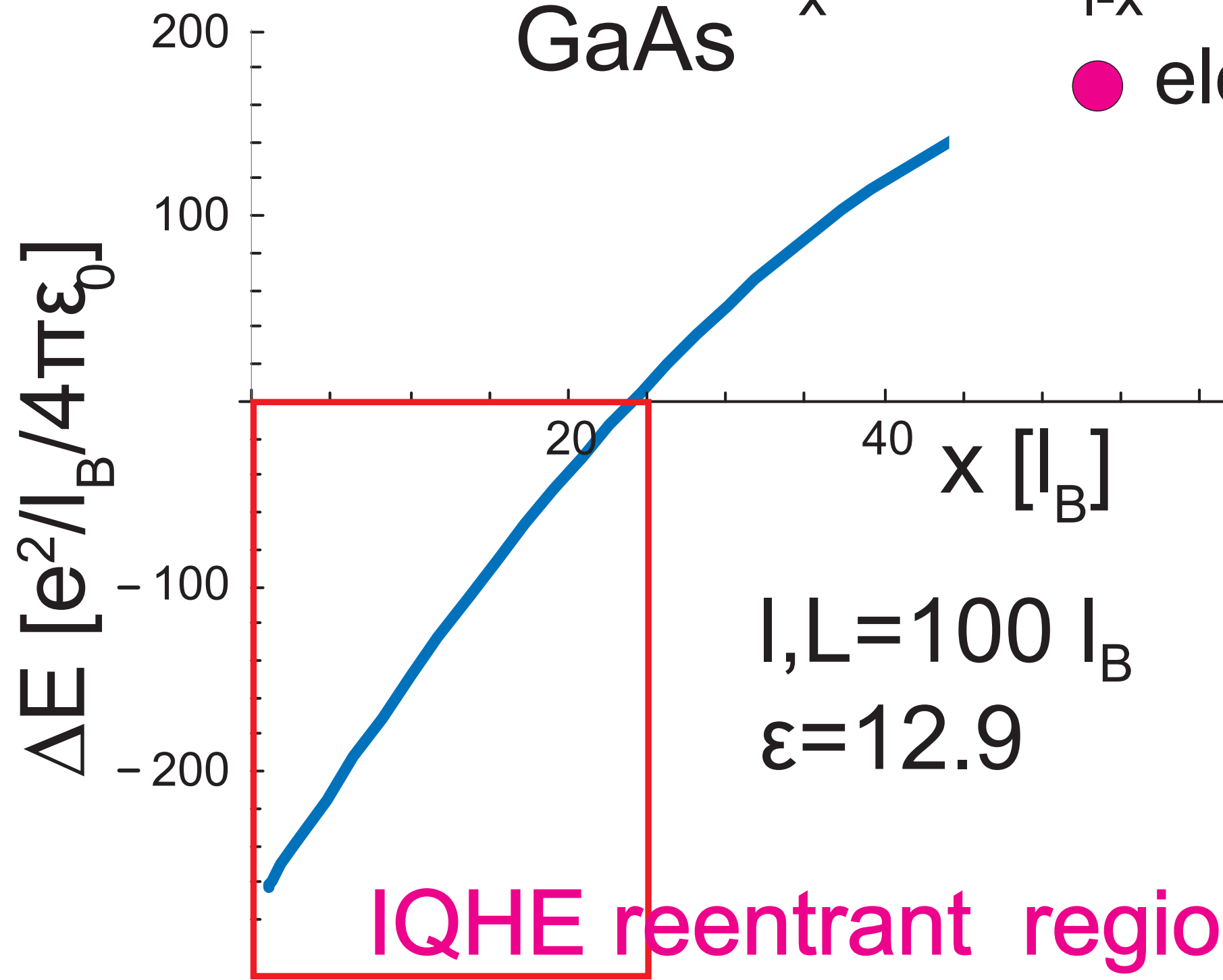

Supplement: Supplementary file 1 [file nanomaterials-10-01286-s001.zip › Definitions/wg1000-eps-converted-to.pdf]

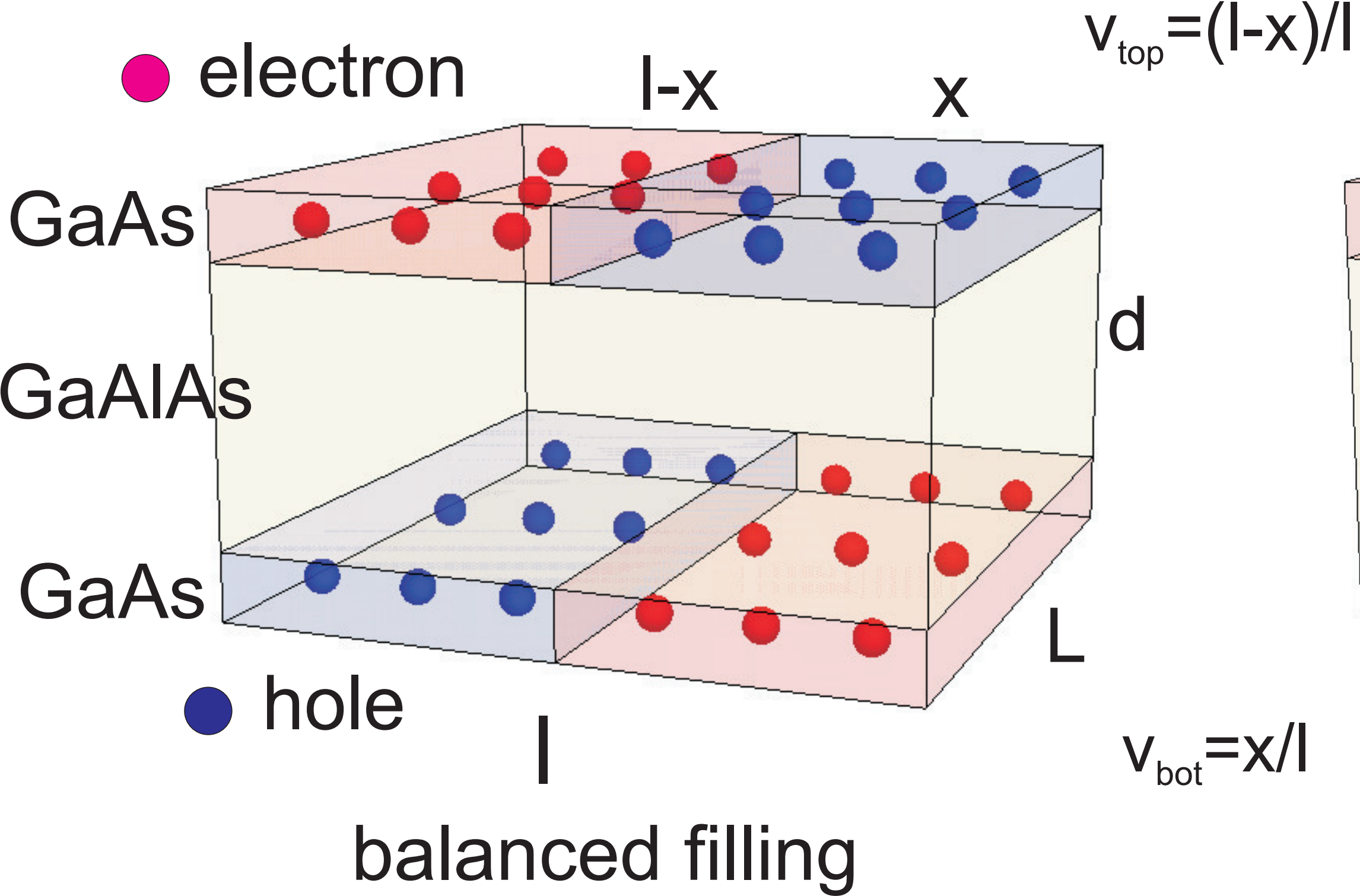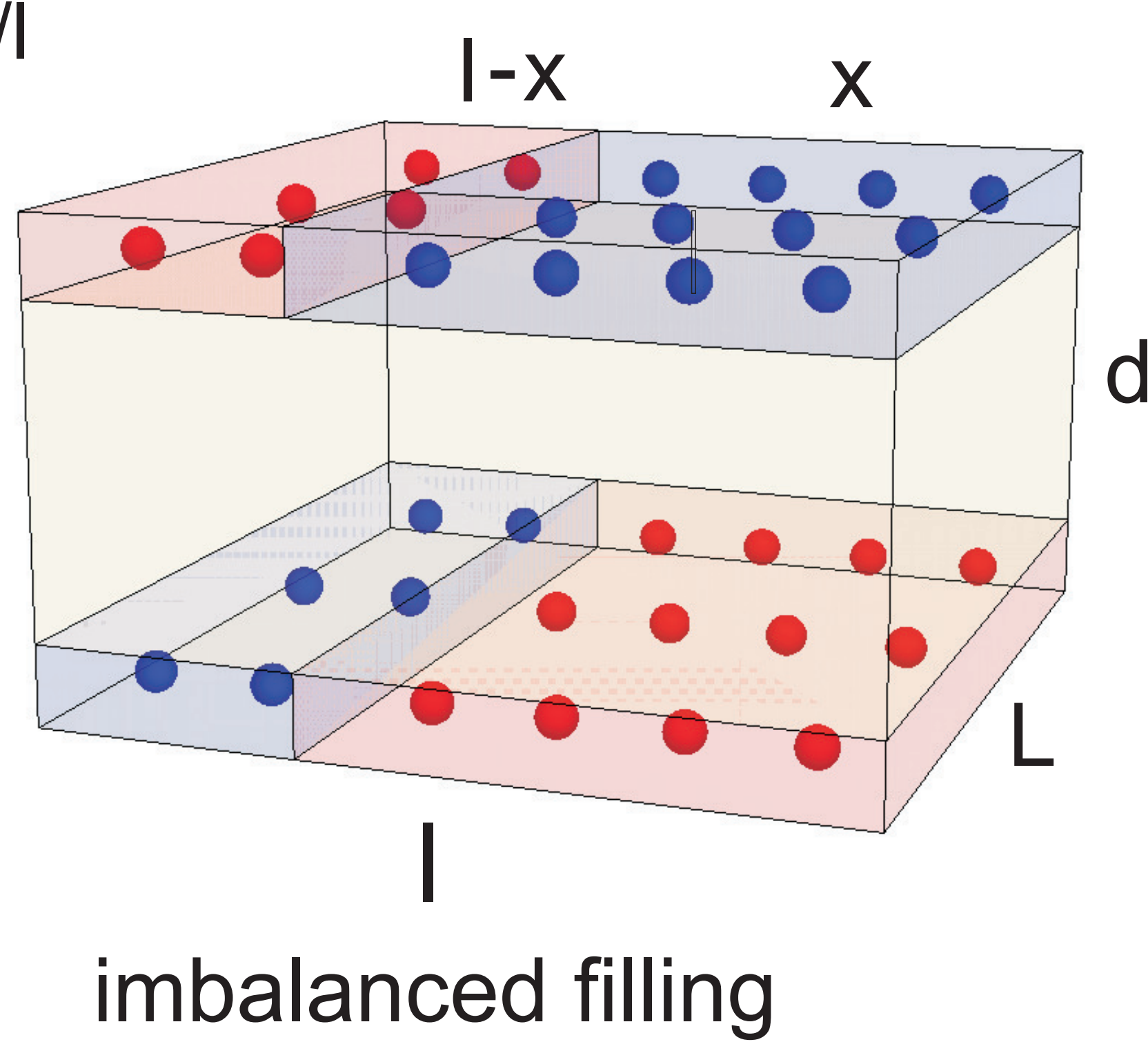

Supplement: Supplementary file 1 [file nanomaterials-10-01286-s001.zip › Definitions/wg600-eps-converted-to.pdf]
